# A Lipophilic IR-780 Dye-Encapsulated Zwitterionic Polymer-Lipid Micellar Nanoparticle for Enhanced Photothermal Therapy and NIR-Based Fluorescence Imaging in a Cervical Tumor Mouse Model

**DOI:** 10.3390/ijms19041189

**Published:** 2018-04-13

**Authors:** Santhosh Kalash Rajendrakumar, Ning-Chu Chang, Adityanarayan Mohapatra, Saji Uthaman, Byeong-Il Lee, Wei-bor Tsai, In-Kyu Park

**Affiliations:** 1 Department of Biomedical Science and BK21 PLUS Center for Creative Biomedical Scientists at Chonnam National University, Chonnam National University Medical School, Gwangju 61469, Korea; kalash1288@gmail.com (S.K.R.); mkaditya55@gmail.com (A.M.); 2 Department of chemical Engineering, National Taiwan University, Taipei 10617, Taiwan; r05524045@ntu.edu.tw; 3 Department of Polymer Science and Engineering, Chungnam National University, 99 Daehak-ro, Yuseong-gu, Daejeon 34134, Korea; sajiuthaman@gmail.com; 4 Medical Photonics Research Center, Korea Photonics Technology Institute, Gwangju 61007, Korea; bilee@kopti.re.kr

**Keywords:** micelle, hydrophobic, IR-780, near-infrared, imaging, cervical cancer, photothermal therapy, zwitterion

## Abstract

To prolong blood circulation and avoid the triggering of immune responses, nanoparticles in the bloodstream require conjugation with polyethylene glycol (PEG). However, PEGylation hinders the interaction between the nanoparticles and the tumor cells and therefore limits the applications of PEGylated nanoparticles for therapeutic drug delivery. To overcome this limitation, zwitterionic materials can be used to enhance the systemic blood circulation and tumor-specific delivery of hydrophobic agents such as IR-780 iodide dye for photothermal therapy. Herein, we developed micellar nanoparticles using the amphiphilic homopolymer poly(12-(methacryloyloxy)dodecyl phosphorylcholine) (PCB-lipid) synthesized via reversible addition–fragmentation chain transfer (RAFT) polymerization. The PCB-lipid can self-assemble into micelles and encapsulate IR-780 dye (PCB-lipid–IR-780). Our results demonstrated that PCB-lipid–IR-780 nanoparticle (NP) exhibited low cytotoxicity and remarkable photothermal cytotoxicity to cervical cancer cells (TC-1) upon near-infrared (NIR) laser irradiation. The biodistribution of PCB-lipid–IR-780 showed higher accumulation of PCB-lipid–IR-780 than that of free IR-780 in the TC-1 tumor. Furthermore, following NIR laser irradiation of the tumor region, the PCB-lipid–IR-780 accumulated in the tumor facilitated enhanced tumor ablation and subsequent tumor regression in the TC-1 xenograft model. Hence, these zwitterionic polymer-lipid hybrid micellar nanoparticles show great potential for cancer theranostics and might be beneficial for clinical applications.

## 1. Introduction

Traditional therapies for cancer such as surgery, chemotherapy, and radiotherapy are invasive and nonselective treatment strategies that have notable side effects and poor treatment outcomes [1]. Hence, alternative treatment methods such as photothermal or photodynamic therapies show enhanced spatiotemporal selectivity and minimal invasiveness [2]. Photothermal therapy (PTT) uses photoabsorbing materials that generate heat for tumor ablation upon near-infrared (NIR) laser irradiation [2]. The increased heat generation above 49 °C degrades functional proteins and thus induces necrosis and apoptosis in the cancer cells [2]. Dye-based NIR-absorbing nanomaterials have theranostic properties and are used in NIR imaging and hyperthermic tumor ablation. IR-780 dye is a hydrophobic heptamethine dye with higher fluorescent intensity than the Food and Drug Administration (FDA)-approved indocyanine green (ICG) dye, although it lacks solubility in aqueous solution and is eliminated more rapidly from the body system [3]. Hence, encapsulation of the IR-780 dye in amphiphilic micelle nanoparticles is expected to enhance its stability in aqueous solutions [4,5,6]. Ye Kuang et al. encapsulated IR-780 dye in cyclic RGD-conjugated solid lipid nanoparticles and elucidated the enhanced NIR live-image-guided photothermal ablation of U87MG tumors [7].

**Figure 1:**
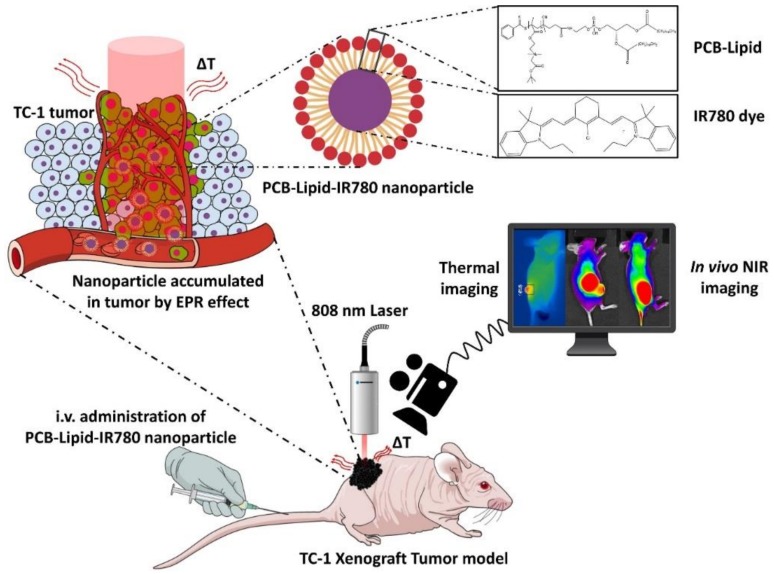
Schematic representation of PCB-lipid–IR-780 nanoparticles for photothermal ablation in a TC-1 xenograft tumor model. Intravenously injected PCB-lipid–IR-780 nanoparticles accumulate in the TC-1 tumor due to the enhanced permeability and retention (EPR) effect. Later accumulation of nanoparticles was monitored concomitantly with an in vivo near-infrared (NIR) imaging system, and the 808-nm laser irradiated the tumor region to ablate the target tumor. The heat generation (ΔT) was monitored using a thermal imaging camera.

Micellar nanoparticles can be developed with a wide range of sizes and surface charges. In most of the developed micellar nanoparticles, the size is less than 100 nm to limit the liver accumulation during systemic circulation [8]. However, the surface charge of the nanoparticles (either positive or negative charges) could lead to formation of a protein corona, which causes accumulation of the nanoparticles in the liver [9]. Protein corona formation on nanoparticles can be avoided using PEGylated nanoparticles, therefore improving blood circulation and avoiding liver accumulation of nanoparticles. However, PEGylation hinders the interaction of the nanoparticles with the tumor and thus reduces the tumor uptake of nanoparticles. Hence, as an alternative for enhanced systemic circulation and tumor accumulation, zwitterionic micellar nanoparticles have been developed and widely studied. Previously, Chen et al. [10] developed a lipid-mimicking zwitterionic homopolymer via RAFT polymerization that formed self-assembled nanoparticles loaded with IR-780 dye [10]. The nanoparticles exhibited enhanced tumor accumulation via an enhanced permeability and retention (EPR) effect and displayed the benefits of a zwitterionic phosphorylcholine surface for long-term circulation in the blood system of the body [10]. Herein, we synthesized and studied zwitterionic polymer-lipid hybrid micellar nanoparticles using homopolymer poly(12-(methacryloyloxy)dodecyl phosphorylcholine)-loaded IR-780 dye nanoparticle (PCB-lipid–IR-780 NP) for in vivo photothermal therapy in a TC-1 cervical cancer xenograft tumor model (Figure 1).

## 2. Results

### 2.1. Synthesis and Characterization of PCB-Lipid Micellar Nanoparticles

**Figure 2:**
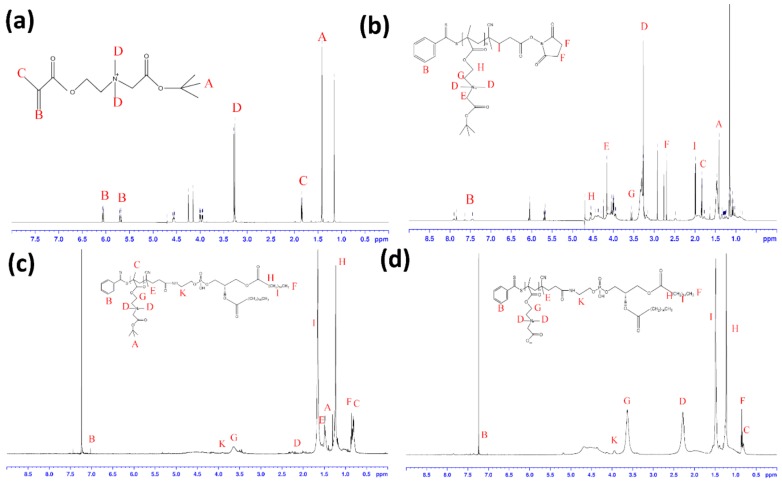
Synthesis of DPPE-PCB (PCB-lipid) by RAFT polymerization. The ^1^H NMR spectrum of (**a**) CB-tBu monomer, (**b**) PCB-tBu, (**c**) DPPE-PCB-tBu, and (**d**) DPPE-PCB.

The CB-tBu monomer was analyzed by ^1^H NMR (D_2_O) δ (ppm): 1.42 (–OC(CH_3_)_3_), 1.84 (CH_2_=C(CH_3_)COO–), 3.26 (–CH_2_N(CH_3_)_2_CH_2_COO–), 3.98 (2H, CH_2_=C(CH_3_)COOCH_2_CH_2_N(CH_3_)_2_CH_2_–), 4.20 (–CH_2_N(CH_3_)_2_CH_2_COO–), 4.56 (2H, CH_2_=C(CH_3_)COOCH_2_CH_2_N(CH_3_)_2_CH_2_–), 5.69 and 6.07 (CH_2_=C(CH_3_)COO–) ^1^H NMR (500 MHz, D_2_O) δ (ppm): 1.42 (s, 9H, –OC(CH_3_)_3_), 1.84 (s, 3H, CH_2_=C(CH_3_)COO–), 3.26 (s, 6H, –CH_2_N(CH_3_)_2_CH_2_COO–), 3.98 (t, *J* = 3 Hz, 2H, CH_2_=C(CH_3_)COOCH_2_CH_2_N(CH_3_)_2_CH_2_–), 4.20 (s, 2H, –CH_2_N(CH_3_)_2_CH_2_COO–), 4.56 (t, *J* = 3 Hz, 2H, CH_2_=C(CH_3_)COOCH_2_CH_2_N(CH_3_)_2_CH_2_–), 5.69 and 6.07 (s, 2H, CH_2_=C(CH_3_)COO–) (Figure 2a), similar to the spectrum shown in previous literature [11].

After polymerization of PCB-tBu via RAFT, the structure of PCB-tBu was confirmed by the ^1^H NMR spectrum (Figure 2b). ^1^H NMR (500 MHz, D_2_O) δ (ppm): 3.3 (–CH_2_N–(CH_3_)_2_CH_2_COO– from CB polymer), 4.16 and 4.56 (–COOCH_2_CH_2_N(CH_3_)_2_CH_2_COO– from CB polymer), 2.64 (–COCH_2_CH_2_CO– from chain transfer agent), 7.45, 7.62 and 7.83 (aromatic ring from chain transfer agent). Schematically, Figure S1 showed NHS–PCB conjugation with DPPE in which the ^1^H NMR (CDCl_3_) of DPPE-PCB-tBu appears (Figure 2c): δ (ppm): 0.85 (t, *J* = 6.7 Hz, 6 H, terminal CH from DPPE), 1.25 (br, s, 48 H, methylene from DPPE), 1.65 (m, 4 H, CH_2_CH_2_C=O from DPPE), 1.49(s, 9H, –OC(CH_3_)_3_ from PCB-tBu), 7.00 and 7.45 (aromatic ring from PCB-tBu).

After treatment with TFA for 4 h, the t-butyl ester group of DPPE-PCB-tBu was removed and became DPPE-PCB. The ^1^H NMR (500 MHz, CDCl_3_) of DPPE-PCB is shown in Figure 2d. Compared with the ^1^H NMR spectrum of DPPE-PCB-tBu, the peak for the t-butyl ester group at δ = 1.49 ppm disappeared in the ^1^H NMR spectrum of DPPE-PCB, confirming the removal of the t-butyl ester group.

### 2.2. Synthesis and Characterization of PCB-Lipid–IR-780 Nanoparticles

**Figure 3:**
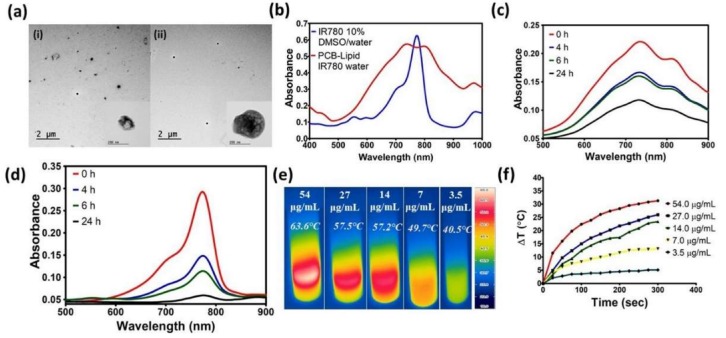
Characterization of PCB-lipid–IR-780 nanoparticles. (**a**) TEM image of (i) PCB-lipid micelle nanoparticles and (ii) PCB-lipid–IR-780 nanoparticles; (**b**) absorbance spectra of IR-780 in 10% DMSO/water and PCB-lipid–IR-780 NPs in water, and absorbance spectra of (**c**) PCB-lipid–IR-780 NPs and (**d**) free IR-780 dye exposed to daylight at different time points; (**e**) thermal images of PCB-lipid–IR-780 NPs irradiated with 808-nm laser at 2 W/cm^2^ for 5 min; and (**f**) heat curve of PCB-lipid–IR-780 NPs irradiated with 808-nm laser at 2 W/cm^2^ for 5 min.

Due to the amphiphilic nature of the PCB-lipid, it can self-assemble into micellar nanoparticles and therefore encapsulate the lipophilic IR-780 dye into the hydrophobic core. The TEM and DLS characterization of the PCB-lipid–IR-780 nanoparticles showed a size of 318 nm with PDI < 0.1 (Figure 3a and Figure S2). The Loading Capacity (%) and Encapsulation Efficiency (%) of the PCB-lipid–IR-780 were determined as 5.1 and 51.1, respectively. The absorbance spectra of IR-780 alone and of PCB-lipid IR-780 micelles dissolved in DMSO/ethanol cosolvent displayed a strong absorption at 788 nm, although IR-780 dissolved in aqueous solution mixed with 10% DMSO and PCB-lipid IR-780 micelles in water had absorption peaks with different extents of red or blueshifts located at 704, 734, and 803 nm, respectively (Figure 3b).

Later, we investigated the photostability of PCB-lipid–IR-780 micelles using the UV–vis spectra of both daylight-exposed free IR-780 solution and PCB-lipid–IR-780 micelles after different time intervals (Figure 3c,d). As shown in Figure 3d, after a 4 h exposure, free IR-780 showed a drastic reduction in absorbance, whereas PCB-lipid–IR-780 displayed a slight decline in the absorbance. However, after a 24 h exposure, PCB-lipid–IR-780 micelles still maintained approximately 50% of the maximum absorbance. In the case of free IR-780 solution, the maximum absorbance decreased to the baseline level. These results demonstrated that PCB-lipid–IR-780 micelles could offer excellent antiphotobleaching capability and are thus suitable for further animal experiments.

With 808-nm laser irradiation at 2 W/cm^2^ for 5 min, the temperature changes of PCB-lipid–IR-780 were observed with respect to different IR-780 concentrations (µg/mL). At 7 µg/mL IR-780, the temperature increased above the photothermal lethal dose (>49 °C) with a 5-min irradiation time (Figure 3e,f). This result suggests that for in vivo experiments, low concentrations of IR-780 must be accumulated in the tumor for photothermal ablation.

### 2.3. Photothermal-Mediated Toxicity to NIR Laser-Irradiated TC-1 Cancer Cells Treated with PCB-Lipid–IR-780 Nanoparticles

**Figure 4:**
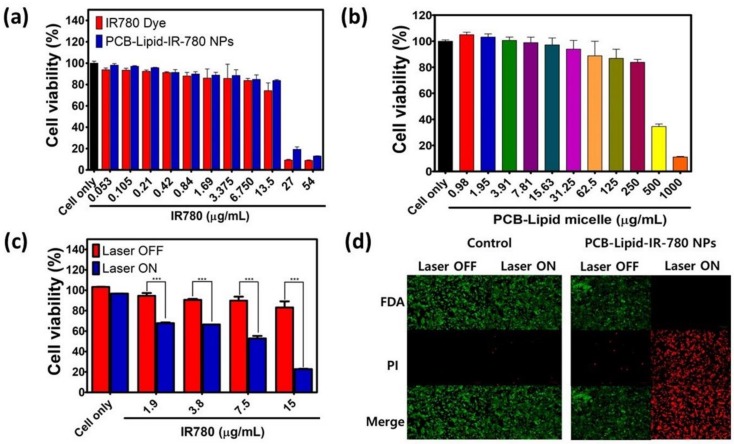
Cytotoxicity of PCB-lipid–IR-780-NP-treated TC-1 cells postirradiation with NIR laser. (**a**) Cell viability of TC-1 cells treated with PCB-lipid–IR-780 NPs at varying concentrations of IR-780; (**b**) cell viability of TC-1 cells treated with different concentrations of PCB-lipid micellar NPs; (**c**) cell viability of TC-1 cells treated with PCB-lipid–IR-780 NPs at varying concentrations of IR-780 and irradiated with 808-nm laser at 2 W/cm^2^ for 5 min; and (**d**) live/dead cell assay of TC-1 treated with PCB-lipid–IR-780 NPs and irradiated with 808-nm laser at 2 W/cm^2^ for 5 min. FDA: fluorescein diacetate; PI: propidium iodide. *n* = 4, SEM, *** *p* ≤ 0.001.

Before assessing the photothermal-mediated toxicity to TC-1 cells, it is necessary to investigate the viability of TC-1 cells treated with PCB-lipid–IR-780 nanoparticles at varied concentrations. Figure 4a,b shows that TC-1 cells treated with different concentrations of PCB-lipid–IR-780 based on the loaded IR-780 displayed greater viability than those treated with free IR-780 dye up to 13.5 μg/mL, and therefore was considered nontoxic. Similarly, PCB-lipid–IR-780 was also nontoxic to the RAW264.7 cell line (Figure S4). TC-1 cells treated with different concentrations of IR-780 in PCB-lipid were viable prior to laser irradiation. However, upon 808-nm laser irradiation, TC-1 cells showed a substantial reduction in cell viability with respect to increasing PCB-lipid–IR-780 concentration (Figure 4c). This result emphasized that the cytotoxicity of laser irradiation on PCB-lipid–IR-780-treated TC-1 cells was concentration-dependent. Additionally, the live/dead assay demonstrated that local irradiation of PCB-lipid–IR-780-NP-treated TC-1 cells with the 808-nm laser at 2 W/cm^2^ resulted in high cell death, as shown by the red fluorescence, whereas the treatment of PCB-lipid–IR-780 NPs in TC-1 cells without laser irradiation and laser irradiation on nontreated cells did not cause any sign of cell death, and the cells were viable, as shown by the green fluorescence (Figure 4d).

### 2.4. Intracellular Uptake of PCB-Lipid–IR-780 Nanoparticles by TC-1 Cell Line

**Figure 5:**
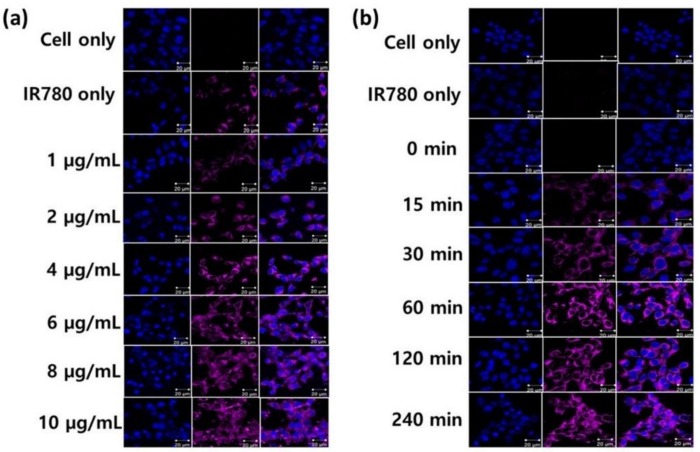
Intracellular uptake of PCB-lipid–IR-780 NPs in the TC-1 cell line. (**a**) NIR fluorescent image of TC-1 cells treated with PCB-lipid–IR-780 NPs at different concentrations and (**b**) NIR fluorescent image of TC-1 cells treated with PCB-lipid–IR-780 NPs (8 µg/mL IR-780) at different time points. Scale bar, 20 µm.

TC-1 cells were cultured to assess the intracellular uptake of PCB-lipid–IR-780 nanoparticles at different concentrations and time points. The nuclei of the cells were stained with DAPI. The fixed cells were observed by confocal microscopy, where DAPI was excited at 406 nm and PCB-lipid–IR-780 was excited at 633 nm. As shown in Figure 5a, an increase in the violet fluorescence signal of the PCB-lipid–IR-780 NPs was observed with respect to increasing concentration of IR-780 in the PCB-lipid–IR-780 NPs. However, the TC-1 cells treated with IR-780 dye alone at 5 µg/mL showed little violet fluorescence intensity compared with the cells treated with PCB-lipid–IR-780 NPs at a concentration of 4 µg/mL IR-780. The time point study revealed that the fluorescence intensity of TC-1 cells incubated with the PCB-lipid–IR-780 NPs was enhanced at postincubation with PCB-lipid–IR-780 NPs for 60 min, as shown in Figure 5b. The intracellular intensity of IR-780 dye in the TC-1 cells was increased with respect to the coincubation time.

### 2.5. Biodistribution of PCB-Lipid–IR-780 Nanoparticles in TC-1 Xenograft Tumor Model

**Figure 6:**
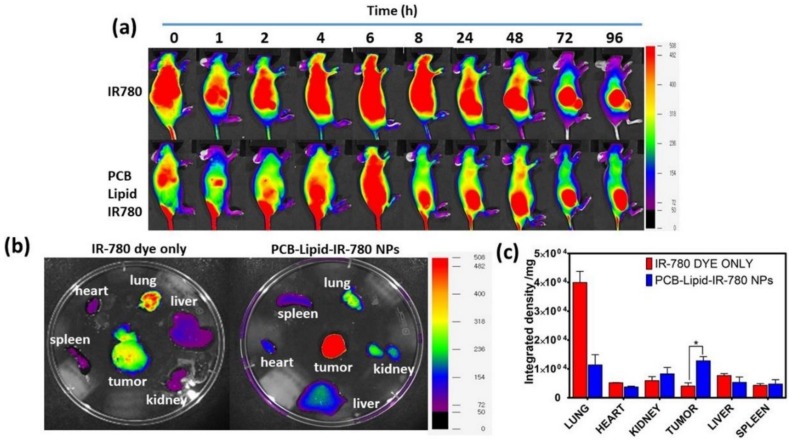
Biodistribution of PCB-lipid–IR-780 NPs in a TC-1 xenograft tumor model. (**a**) NIR fluorescent image of PCB-lipid–IR-780 NPs injected into TC-1 tumor mice at different time points; (**b**) ex vivo NIR fluorescent images of the organs isolated from IR-780 and PCB-lipid–IR-780-NP-treated TC-1 tumor-bearing mice at 48 h postinjection; and (**c**) integrated density plot of the excised organs from IR-780 and PCB-lipid–IR-780-NP-treated TC-1 tumor-bearing mice. *n* = 4, SEM, * *p* ≤ 0.05.

TC-1 xenograft nude mice were injected intravenously with 2 mg/kg of IR-780 in 100 µL of PBS. At the 8-h time point, PCB-lipid–IR-780-treated mice showed an NIR fluorescence localized specifically in the tumor, whereas IR-780-alone-treated mice displayed NIR fluorescence distributed initially throughout the whole body with a continuous reduction in fluorescence intensity, and the injected IR-780 was localized in the tumor at the later stage of 72 h postinjection (Figure 6a). The ex vivo fluorescent images of the organs taken at the 24-h time point demonstrated that PCB-lipid–IR-780 was localized more specifically, with enhanced fluorescent intensity at the tumor site following intravenous (IV) injection compared with IR-780 alone (Figure 6b). The IV administration of IR-780 alone exhibited an initial strong signal in the lung, whereas the PCB-lipid–IR-780-treated group had greatly reduced intensity in the lung (Figure 6c).

### 2.6. Antitumor Efficacy of PCB-Lipid–IR-780 Nanoparticles in TC-1 Xenograft Tumor Model after NIR Laser Irradiation

**Figure 7:**
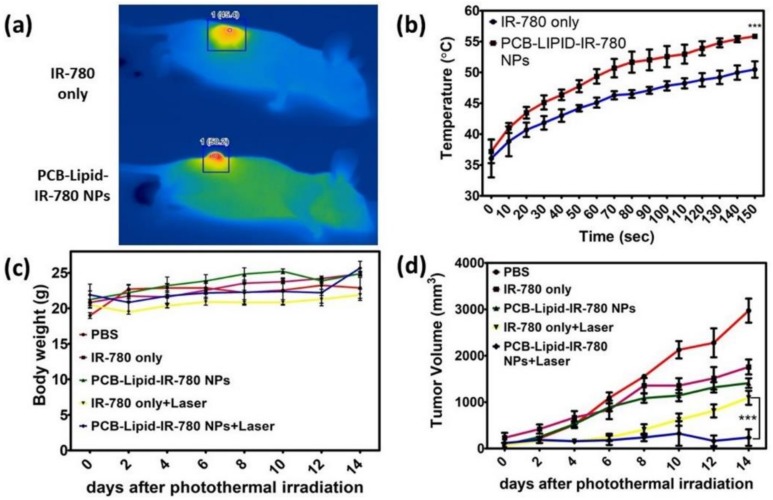
Photothermal-mediated antitumor effect of PCB-lipid–IR-780 NPs in the TC-1 xenograft tumor-bearing model. (**a**) Thermal image of laser-irradiated TC-1 tumor in IR-780 and PCB-lipid–IR-780 treated tumor mice; (**b**) heat curve of laser-irradiated TC-1 tumors in IR-780 and PCB-lipid–IR-780 treated tumor mice; (**c**) whole body weight of laser-irradiated TC-1 tumor mice and (**d**) tumor volume of TC-1 tumor irradiated with 808-nm laser at 2 W/cm^2^ for 2.5 min. *n* = 4, SEM, *** *p* ≤ 0.001.

Laser irradiation was performed at 24 h after intravenous injection of PCB-lipid–IR-780 NPs in TC-1 tumor-bearing mice. Following laser irradiation of the tumor of IR-780-alone-treated mice, the temperature reached 45.4 °C after 5 min of irradiation with the 808-nm laser at 2 W/cm^2^, whereas the temperature of the PCB-lipid–IR-780 group tumor was further elevated to 50.2 °C (Figure 7a,b). After laser irradiation, the tumor volume and body weight of the mice were measured for up to 14 days (Figure 7c). As shown in Figure 7d and Figure S5, the IR-780 and PCB-lipid–IR-780-treated groups showed a significant reduction in the tumor volume at the initial stage compared with the PBS control mice. Although the laser-irradiated groups of IR-780 and PCB-lipid–IR-780 showed significant drastic reduction in the tumor volume compared with the nonirradiated tumors, the sample-treated tumor-bearing mice did not show any reduction in body weight for up to 14 days of observation.

**Figure 8:**
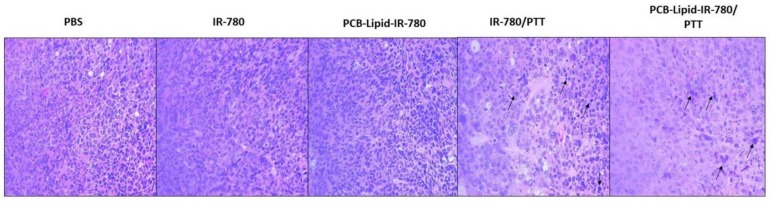
H&E staining on PCB-lipid–IR-780-treated and laser-irradiated TC-1 tumor tissue.

After 14 days, the organs and tumor were isolated and subjected to H&E staining (Figure 8). After laser irradiation, cancer cells undergo apoptosis, and during that process, chromatin changes from a heterogeneous condition to a highly condensed form. The tumor section from the PCB-lipid–IR-780-nanoparticle-treated laser-irradiated group showed a condensed nucleus and voids surrounding the cancer cells, indicating the presence of residual apoptotic cancer cells. Additionally, compared with the IR-780 laser-irradiated group, the PCB-lipid–IR-780-treated group displayed a higher number of necrotic cells. In Figure S6, the result also showed that H&E staining of the liver, lung, and kidney obtained from the PCB-lipid–IR-780-treated and laser-irradiated tumor-bearing mice indicated no significant toxicity compared with the IR-780-treated and laser-irradiated group.

## 3. Discussion

The PCB-lipid was synthesized via RAFT polymerization and was confirmed by ^1^H NMR. Previously, Chen et al. developed a similar zwitterionic phospholipid-mimicking micellar nanoparticle for NIR imaging and PTT, although the in vivo efficacy of the nanoparticles was not elucidated [10]. Hence, in this work, we synthesized the zwitterionic PCB-lipid–IR-780 NPs for imaging and high-efficiency PTT in a cervical tumor model. The size of the nanoparticles is less than 500 nm, with a net negative surface charge of −0.2 mV (Figure 3a). Additionally, the critical micellar concentration (CMC) of the PCB-lipid was 127 µg/mL, suggesting that PCB-lipid micelles were formed at low concentration and could load hydrophobic drugs more efficiently (Figure S3). The IR-780–PCB-lipid feed ratio was set to a 1:10 weight ratio, and therefore, the LC (%) and EE (%) values were obtained as 5.1 and 51.1, respectively, and they are comparatively higher than other micellar nanoparticle for IR-780 delivery [12,13]. The absorbance spectrum of the IR-780 dye loaded in the PCB-lipid–IR-780 NPs showed a 15-nm redshift to 803 nm, indicating that hydrophobic dye inside the micelles experienced a change in polarity and hydrophobic interactions inside the micelles; and such a redshift also demonstrated that IR-780 was efficiently loaded into the PCB-lipid micellar NPs rather than physically adsorbed onto the surface of the nanoparticles [7,14,15]. Bare fluorescent dyes are usually prone to photolysis after exposure to daylight, although it has been shown previously that micellar nanoparticles can protect dyes from photolysis [16,17,18,19]. Wang et al. reported that pullulan-coated phospholipid and pluronic F68-complexed nanoparticles efficiently protected IR-780 dye from daylight-triggered degradation [20]. Similarly, in this work, PCB-lipid–IR-780 NPs also protected IR-780 from daylight more efficiently than bare IR-780 alone for up to 24 h (Figure 3c,d). IR-780 has strong absorbance at 780 nm and is considered an efficient PTT agent for cancer therapy [21]. It was also shown in our study that upon 808-nm laser irradiation, the temperature curve of the PCB-lipid–IR-780 NPs increased with respect to increasing IR-780 concentration and irradiation time (Figure 3e,f). In previously developed IR-780-loaded nanoparticles [7,10,22], temperature differences (ΔT > 10) were achieved with IR-780 concentration at 50 μg or above, whereas the highly stable PCB-lipid–IR-780 NPs showed a similar heating temperature profile at a lower IR-780 concentration of 7 µg/mL in the NPs.

IR-780 dyes are hydrophobic and therefore exert a nonspecific toxic effect over both healthy and cancerous cells [23]. Hence, the performed cytotoxicity assay demonstrated that PCB-lipid–IR-780 nanoparticles did not show any significant toxicity to TC-1 and RAW264.7 cell lines at up to 15 µg/mL IR-780 cell concentration compared with bare IR-780 dye. In addition, the micellar nanoparticles alone also exerted no noticeable cytotoxicity, even up to 250 µg/mL concentration. Thus, these results signify that PCB-lipid–IR-780 nanoparticles are safe and can be administered in an animal model for safe and efficient delivery of IR-780 to the tumor site. However, the cytotoxic effect of PCB-lipid–IR-780 nanoparticles was observed in the TC-1 cell line only after NIR laser irradiation. The viability of the cells decreased with increasing NP concentration. An increase in temperature of up to 43 °C is required to denature more than 5% of total cellular proteins, which initiates apoptosis in cancer cells [24]. According to the thermal curve results, the rise in the intracellular temperature to above 43 °C depends proportionally on the local concentration of IR-780 dye in the irradiated region. In this work, the killing effect of PCB-lipid–IR-780 nanoparticles in TC-1 cells was due to increasing IR-780 concentration as well as the temperature rise above 43 °C after 808-nm laser irradiation. The results from the live/dead assay also supported the hypothesis that cell death occurred only after laser irradiation in the PCB-lipid–IR-780-nanoparticle-treated TC-1 cells and was absent in the nonirradiated or laser-irradiated nontreated TC-1 cells. Enhanced photothermal killing of TC-1 cells can also be attributed to the enhanced intracellular uptake of PCB-lipid–IR-780 nanoparticles. Zwitterionic-based nanoparticles are not efficient in improving the cellular uptake due to poor interaction between the neutrally charged nanoparticle and the cancer cell membrane [25]. However, the intracellular uptake study revealed that intracellular accumulation of IR-780 dye was facilitated with increasing concentration of PCB-lipid–IR-780 nanoparticles compared with bare IR-780, showing enhanced uptake of the nanoparticles in the TC-1 cells within 60 min. This observation can be explained by the fact that amphilic polymers have the ability to overcome the cell membrane barrier and efficiently deliver the cargo to the cytosol [26,27].

Previously, it was reported by Arvizo et al. that the surface charge of the nanoparticles influences the biodistribution and tumor uptake [28]. In that study, the zwitterionic nanoparticle showed low plasma clearance after administration via intravenous injection [28]. In this work, biodistribution analysis of PCB-lipid–IR-780 nanoparticles in a TC-1 xenograft model was performed. IR-780 dye alone injected into the tumor mice displayed significant accumulation in the lung at 48 h postinjection, whereas the PCB-lipid–IR-780 nanoparticles achieved significantly enhanced accumulation in the tumor at the same time. The NIR images of ex vivo organs also suggested the same pattern. Compared with mice injected with IR-780 dye alone, the PCB-lipid–IR-780-nanoparticle-injected group attained a significant increase in accumulation at the tumor site. The long-term systemic circulation of the PCB-lipid–IR-780 nanoparticles can be attributed to the zwitterionic property, whereas the increased tumor accumulation can be attributed partially to the EPR effect.

According to the biodistribution study, the PCB-lipid–IR-780 nanoparticles accumulated completely in the tumor site at the 48-h time point following IV injection, and hence, NIR laser irradiation treatment was performed at that time. The thermal images of the laser-irradiated tumor sites of the IR-780-injected mice group and the PCB-lipid–IR-780 nanoparticle-injected group showed that the temperature was significantly increased in the PCB-lipid–IR-780-nanoparticle-injected group compared with the IR-780-alone-injected group. This observation could result from the higher accumulation of PCB-lipid–IR-780 nanoparticles in the tumor compared with that of the bare dye alone. The subsequent antitumor study with NIR laser irradiation of PCB-lipid–IR-780 NPs tumor-bearing mice demonstrated a significant reduction in the tumor volume compared with the nonirradiated tumor mice. The H&E staining of the treated tumors also clearly showed the presence of apoptotic cells with condensed nuclei and more numerous necrotic cells in the PCB-lipid–IR-780-NP-treated and laser-irradiated mice group. Overall, the in vivo study demonstrated that the long-term circulation, EPR effect, and NIR laser irradiation contributed to eradicating the tumor burden in TC-1 tumor-bearing mice. Our results revealed that PCB-lipid–IR-780 NPs could be a safe, highly efficient, and promising agent for in vivo photothermal therapy of TC-1 cervical cancer tumors.

## 4. Materials and Methods

### 4.1. Materials

The 2-(dimethylamino) ethyl methacrylate, tert-butyl bromoacetate (tBu), 4-cyano-4 (phenycarbonothioylthio)pentanoic acid *N*-succinimidyl ester, and IR-780 iodide dye were purchased from Sigma-Aldrich (St. Louis, MS, USA). The 1,2-dipalmitoyl-sn-glycero-3-phosphoethanolamine (DPPE) was purchased from Bachem (Bubendorf, Switzerland). The 2,2′-azodiisobutyronitrile (AIBN) was purchased from UR Biotech (Taipei, Taiwan). The 1,2-distearoyl-sn-glycero-3-phosphocholine (DSPC) was purchased from Avanti (Alabaster, AL, USA).

### 4.2. Synthesis of CB-tBu Monomer

The synthetic procedure for CB-tBu was performed, according to the literature [11]. In brief, 2-(dimethylamino)ethyl methacrylate (3.77 g, 24 mmol) and tert-butyl bromoacetate (5.50 g, 28.2 mmol) were mixed in 20 mL acetonitrile (ACN) and allowed to react at 50 °C under a N_2_ environment for 24 h. The reaction was stopped in an ice bath. The mixture was poured into 200 mL diethyl ether, and precipitate formed at 4 °C. The white precipitate was filtered, rinsed with fresh diethyl ether, and dried. The resulting CB-tBu monomers were stored at −20 °C until use (yield 94.5%).

### 4.3. Synthesis of NHS-PCB-tBu *via* RAFT Polymerization

NHS-PCB-tBu was synthesized by reversible addition–fragmentation chain transfer (RAFT) polymerization using 4-cyano-4-(phenylcarbonothioylthio)pentanoic acid *N*-succinimidyl ester as the chain transfer agent and 2,2′-azodiisobutyronitrile (AIBN) as the thermal radical initiator. In brief, 0.65 g CB-tBu, 36 mg 4-cyano-4-(phenylcarbonothioylthio)pentanoic acid *N*-succinimidyl ester, and 3.5 mg AIBN were dissolved in 5 mL *N*,*N*-dimethylformamide (DMF), followed by purging with N_2_ for 1 h in an ice bath. The polymerization was initiated at 70 °C and allowed to continue for 2 h. The polymerization was quenched in an ice bath. The polymer was precipitated in ethyl acetate. The product was collected and dried under vacuum for 24 h at room temperature.

### 4.4. Synthesis of DPPE-PCB

The synthesis of DPPE-PCB began with the conjugation of NHS-PCB-tBu with 1,2-dipalmitoyl-sn-glycero−3-phosphoethanolamine (DPPE), according to the literature [29]. In brief, 0.785 g PCB-tBu and 0.38 g DPPE were dissolved in a mixed solvent containing 60 mL chloroform, 8.6 mL DMF, and 150 μL triethylamine, and the mixture was held at room temperature for 5 days (Figure S1). The reaction mixture was evaporated and precipitated in ethyl ether. The precipitates were extracted by acetonitrile and filtered to remove uncoupled DPPE. The filtrate was dried and treated with trifluoroacetic acid for 4 h to remove the t-butyl ester group of PCB-tBu. The product containing DPPE-PCB and unconjugated PCB was precipitated in ethyl ether and dried in a vacuum. The dried product was dissolved in 0.1 M PBS buffer (pH = 7.4) and ultrafiltrated (30 K MW cutoff, Amicon Ultra-15, Millipore, Billerica, MA, USA) in PBS and later in pure water for a total of 5 times to remove unconjugated PCB. The DPPE-PCB product was lyophilized and stored at −20 °C.

### 4.5. Preparation and Characterization of PCB-Lipid–IR-780 Nanoparticles

A 10-mg amount of PCB-lipid micelles was dissolved in 5 mL of Milli-Q water and sonicated for 2 min with 23% amplitude and a 5-s pulse in an ice bath. During sonication for another 10 min, 1 mg/mL of IR-780 solution in DMSO was added dropwise to the PCB-lipid solution in an ice bath, and the solution was stirred at room temperature overnight. The DMSO was removed via dialysis against an excess amount of distilled water (D.W.) with a MWCO 3500 dialysis bag (Spectra/Por^®^, Rancho Dominguez, CA, USA) for 8 h, and the product was frozen and lyophilized. The encapsulation efficiency (EE) of the micelles was determined, where 1 mg of PCB-lipid–IR-780 was dissolved in a solvent of ethanol and DMSO and the concentration of IR-780 was determined using UV–vis spectroscopy according to the standard curve. The EE and loading efficiency (LE) were calculated based on the formula from previous studies. The photostability of PCB-lipid–IR-780 nanoparticles was determined by analyzing the absorption spectra of PCB-lipid–IR-780 nanoparticles (100 μg/mL PCB-lipid–IR-780) exposed to daylight at different time points using UV–vis spectroscopy. Dynamic light scattering (DLS) size and zeta potential was measured using Zetasizer Nano (Malverin, Worcestershire, UK). The particle size and morphological structure of PCB-Lipid-IR-780 were measured using a field emission TEM (FE-TEM) (Jeol JEM-2100F). The nanoassembly was dried overnight on an amorphous carbon-coated copper grid and then observed at 200 kV.

### 4.6. Critical Micellar Concentration

In vials, 100 μL pyrene in acetone (6 × 10^−7^ M) was added and acetone was evaporated. PCB-lipid micelle in 500 µL D.W. were added to the pyrene containing vials and sonicated for 1 min at 23% amplitude with 5 s pulse. The concentration of micelles ranged from 1 mg/mL to 0.05 mg/mL. After overnight incubation, the emission spectra (*λ*_em_ range = 350–450 nm, *λ*_ex_ = 339 nm) were measured. From the emission spectra, the values of the 373/384 emission peak ratio characteristic of pyrene were plotted as a function of the micelle concentration.

### 4.7. Temperature Measurements under Near-IR Irradiation

The temperature difference (ΔT) of the PCB-lipid–IR-780 during 808-nm laser irradiation was measured with an infrared thermal camera. Different concentrations of PCB-lipid–IR-780 based on IR-780 amount were dissolved in D.W. and was irradiated with 808-nm laser at 2 W/cm^2^ for 5 min. At 30 s intervals, temperatures were recorded and plotted.

### 4.8. Cell Culture and Animal Model

TC-1 and RAW264.7 cell lines were cultured in RPMI 1640 supplemented with 10% FBS, 100 U/mL penicillin, and 100 mg/mL streptomycin at 37 °C in a 5% CO_2_ incubator. The number of cells was counted using an automated cell counter. All experiments involving live animals were performed in compliance with the institutional guidelines of the Chonnam National University Medical School and Chonnam National University Hospital (CNU IACUC-H-2015-47, 25 December 2017), South Korea. Female BALB/c nude mice (5 weeks old) were purchased from Orient Bio Inc., Seongnam-si, South Korea. To set up the tumor model, TC-1 cells (1 × 10^6^) in 100 μL of PBS were subcutaneously injected into the right flank of the mice. Tumor-bearing mice were used in near-IR imaging and photothermal-based antitumor studies when the volume of tumor reached 100 mm, where tumor volume = (tumor length) × (tumor width)^2^/2.

### 4.9. In Vitro Cell Viability Assay using MTS

The in vitro cell cytotoxicity in the RAW264.7 cell line and in TC-1 cells with/without near-IR laser irradiation was evaluated using the MTS cell viability assay. TC-1 or RAW264.7 cells (1 × 10^4^ cells/well) were seeded onto 96-well plates. After overnight incubation, the culture medium was replaced with 100 μL of RPMI 1640 medium containing free IR-780, PCB-lipid micelles or PCB-lipid–IR-780 nanoparticles. After another 24 h incubation, the cell viability was evaluated by MTS assay. Similarly, for the photothermal treatments, TC-1 cells treated with samples at different concentrations were irradiated with a 2 W/cm^2^ 808-nm laser for 5 min. After another 24 h incubation, cell viability was evaluated by MTS assay.

### 4.10. Live and Dead Cell Assay

After irradiating the PCB-lipid–IR-780-NP-treated TC-1 cells with the 808-nm laser at 2 W/cm^2^ for 5 min, the cells were incubated at 37 °C for 1 h. Later, a brief wash with PBS was performed, and the FDA/PI (FDA: fluorescein diacetate, PI: propidium iodide) staining solution in serum-free media was added and incubated for 4 min at room temperature. After washing with PBS, the cells in PBS were directly visualized using ZOE^TM^ fluorescence microscope with green and red filters.

### 4.11. Confocal Microscope Imaging

To determine the intracellular internalization of PCB-lipid–IR-780, TC-1 cells were seeded at 5 × 10^4^ cells per well in Lab-Tek^®^ Chamber Slides and incubated overnight at 37 °C and 5% CO_2_. PCB-lipid–IR-780 samples of different concentrations in OPTI-MEM^®^ were added and incubated for 4 h, followed by aspiration of the media, three washes with 1× PBS, and treatment with 4% paraformaldehyde. DAPI in gold antifade reagent was applied for nuclear staining. Confocal laser scanning microscopy was used to visualize the fluorescence in the samples.

### 4.12. In Vivo Near-IR Imaging and Biodistribution Analysis

After the tumor volume reached 100 mm^3^, the mice were intravenously injected with 100 μL of free IR-780 solution or PCB-lipid–IR-780 (2 mg/kg IR-780 equiv). Whole-body NIR fluorescence images were taken at different time points after injection using the FOBI in vivo imaging system (NeoSciences Co. Ltd., Seoul, South Korea). After cervical dislocation of the tumor mice at 24 h postinjection, the major organs, including heart, liver, spleen, lung, kidney, and tumor, were collected for isolated organ imaging.

### 4.13. In Vivo Photothermal Therapy

The increase in temperature at the tumor region during NIR irradiation in the different experimental groups was evaluated by intravenously injecting PBS, IR-780 (2 mg kg^−1^), and PCB-lipid–IR-780 (2 mg kg^−1^) into mice bearing TC-1 tumors. The temperature changes in the tumor tissues during NIR irradiation (808-nm laser, 2 W/cm^2^ for 2 min) were measured at 24 h postinjection. The region of maximum temperature and the infrared thermographic images were obtained using an infrared thermal imaging camera (Avio IR camera/Thermometer, Shinagawa-ku, Tokyo, Japan).

In vivo photothermal therapy treatment was initiated when the tumor diameter reached 100 mm^3^, with tumor-bearing mice randomly divided into five groups. All samples were administered intravenously via tail-vein injection, and the tumor was exposed to 808-nm NIR laser (2 W/cm^2^) for 2 min at 24 h postinjection (day 0). The tumor size and body weight of each mouse were recorded every three days. The mice were sacrificed on day 18, and lung, liver, kidney, and tumor tissues were harvested. The tissues were fixed in 4% neutral formalin, embedded in paraffin, and sectioned and stained with hematoxylin and eosin (H&E) for histological examination. After H&E staining, the tissues samples were analyzed, and images were acquired on a light microscope.

### 4.14. Statistically Analysis

Statistical analyses were performed using GraphPad Prism 5 (La Jolla, CA, USA). Graphical data are expressed as the average mean ± SEM (standard error of the mean). Two-way ANOVA was used to compare different treatment groups. Differences were considered significant at * *p* ≤ 0.05, and *** *p* ≤ 0.001.

## 5. Conclusions

A zwitterionic polymeric lipid micelle PCB-lipid micelle was synthesized successfully by RAFT polymerization method and further loaded with IR-780 dye for live nanoparticle tracking and photothermal therapy. The fabricated PCB-lipid–IR-780 NPs are highly photostable, biocompatible, and presented an excellent heat conversion upon NIR laser irradiation. Because of the zwitterionic property, a prolonged circulation time and accumulation in the TC-1 tumor was achieved by the PCB-lipid–IR-780 NPs compared to bare IR-780. The photothermal heat induced by laser irradiation has effectively mediated cell death in TC-1 cancer cells as well as in TC-1 tumor xenografts. From our results we can conclude that PCB-lipid–IR-780 NPs holds a great potential for further theranostic application in future clinical trials.

## Supplementary Materials

Supplementary materials can be found at http://www.mdpi.com/1422-0067/19/4/1189/s1.

## Abbreviations

PTT	photothermal therapy
RAFT	reversible addition–fragmentation chain transfer
IR	infrared
NIR	near-infrared
ICG	indocyanine green
FDA	Food and Drug Administration
cRGD	cyclic Arg–Gly–Asp
EPR	enhanced permeability and retention
NMR	nuclear magnetic resonance
DPPE	1,2-dipalmitoyl-sn-glycero-3-phosphoethanolamine
TFA	trifluoroacetic acid
CLSM	confocal laser scanning microscopy
DAPI	4′,6-diamidino-2-phenylindole, dihydrochloride
CMC	critical micellar concentration
RPMI	Roswell Park Memorial Institute medium
FBS	fetal bovine serum
MTS	3-(4,5-dimethylthiazol-2-yl)-5-(3-carboxymethoxyphenyl)-2-(4-sulfophenyl)-2*H*-tetrazolium
SEM	standard error of the mean
ANOVA	analysis of variance
FOBI	fluorescence-labeled organism bioimaging instrument
PBS	phosphate-buffered saline
DMSO	dimethyl sulfoxide
DMF	dimethylformamide
TEM	transmission electron microscopy
AIBN	2,2′-azodiisobutyronitrile
DSPC	1,2-distearoyl-sn-glycero-3-phosphocholine
DLS	Dynamic light scattering
