# Supplementary material for: A Lipophilic IR-780 Dye-Encapsulated Zwitterionic Polymer-Lipid Micellar Nanoparticle for Enhanced Photothermal Therapy and NIR-Based Fluorescence Imaging in a Cervical Tumor Mouse Model
Source: Int J Mol Sci. 2018 Apr 13;19(4):1189. doi: 10.3390/ijms19041189 (PMC5979520; doi:10.3390/ijms19041189)
Supplement: Supplementary file 1 [file ijms-19-01189-s001.pdf]

Supplementary

A Lipophilic IR-780 Dye-Encapsulated Zwitterionic Polymer-Lipid Micellar Nanoparticle for Enhanced Photothermal Therapy and NIR-Based Fluorescence Imaging in a Cervical Tumor Mouse Model

Santhosh Kalash Rajendrakumar, Ning-Chu Chang, Adityanarayan Mohapatra, Saji Uthaman, Byeong-Il Lee, Wei-bor Tsai, and In-Kyu Park

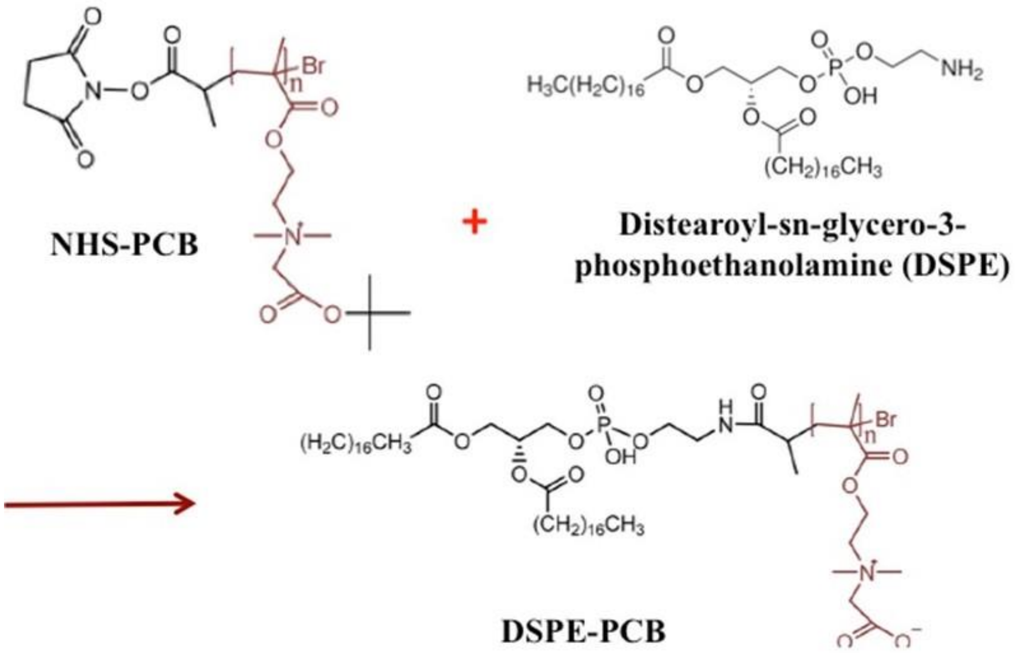

Figure S1. Schematic representation of PCB-lipid synthesis by RAFT polymerization.

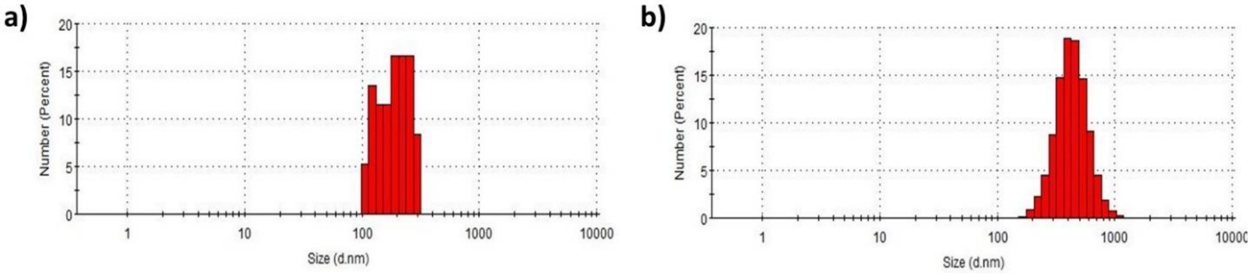

Figure S2. Hydrodynamic size of (**a**) PCB-lipid micellar nanoparticle and (**b**) PCB-lipid–IR-780 in water.

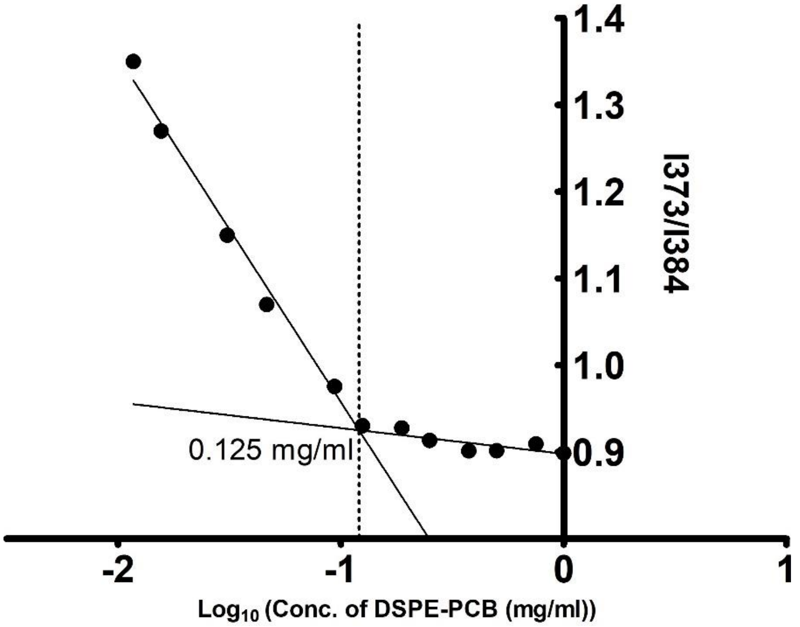

Figure S3. Critical micellar concentration of PCB-lipid micellar nanoparticles.

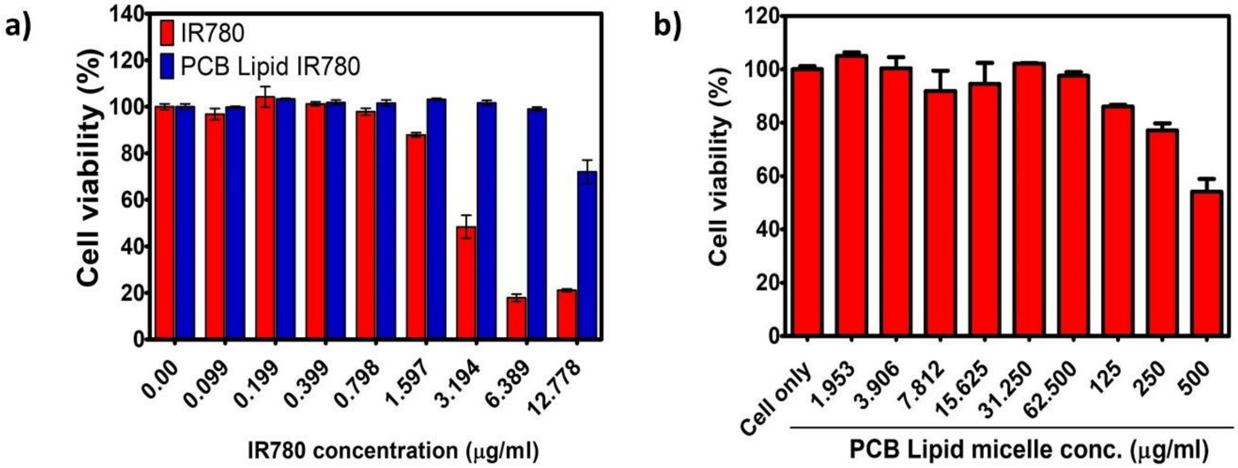

Figure S4. Viability of RAW264.7 cell line treated with PCB-lipid–IR-780 nanoparticles at different IR-780 concentrations. MTS analysis of RAW264.7 cell line treated with (**a**) IR-780 and PCB-lipid–IR-780 NPs; (**b**) PCB-lipid micellar nanoparticles.

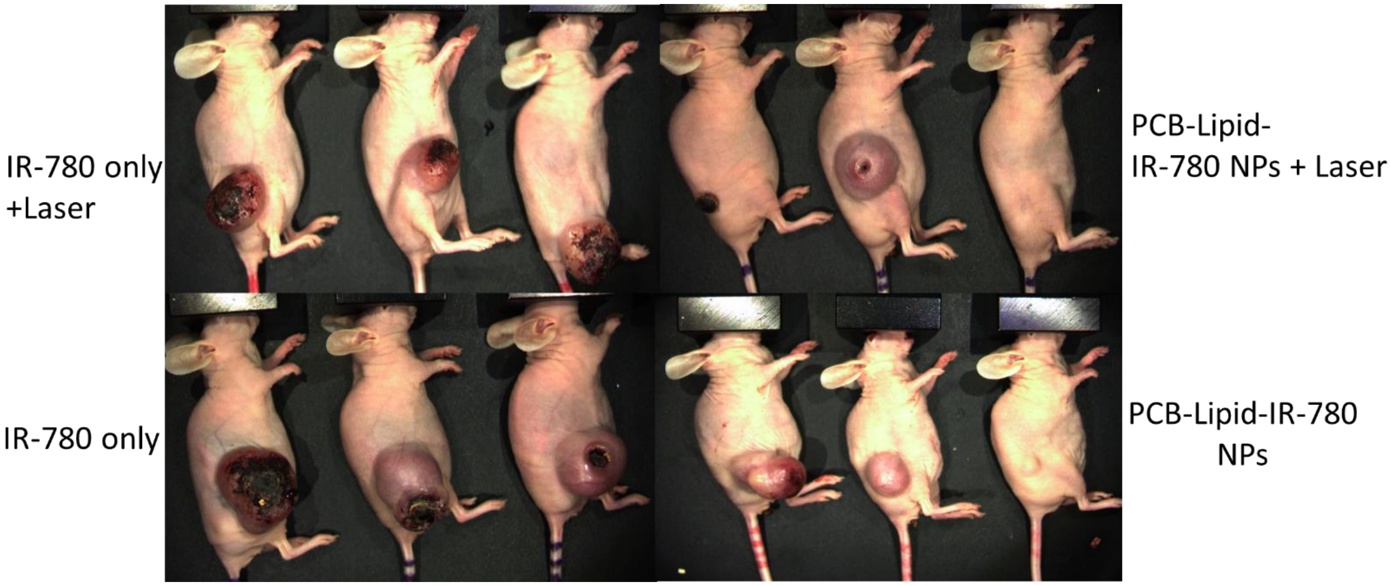

Figure S5. After 16 days, animal images of PTT-treated TC-1 xenograft mouse models administered with PCB-lipid–IR-780.

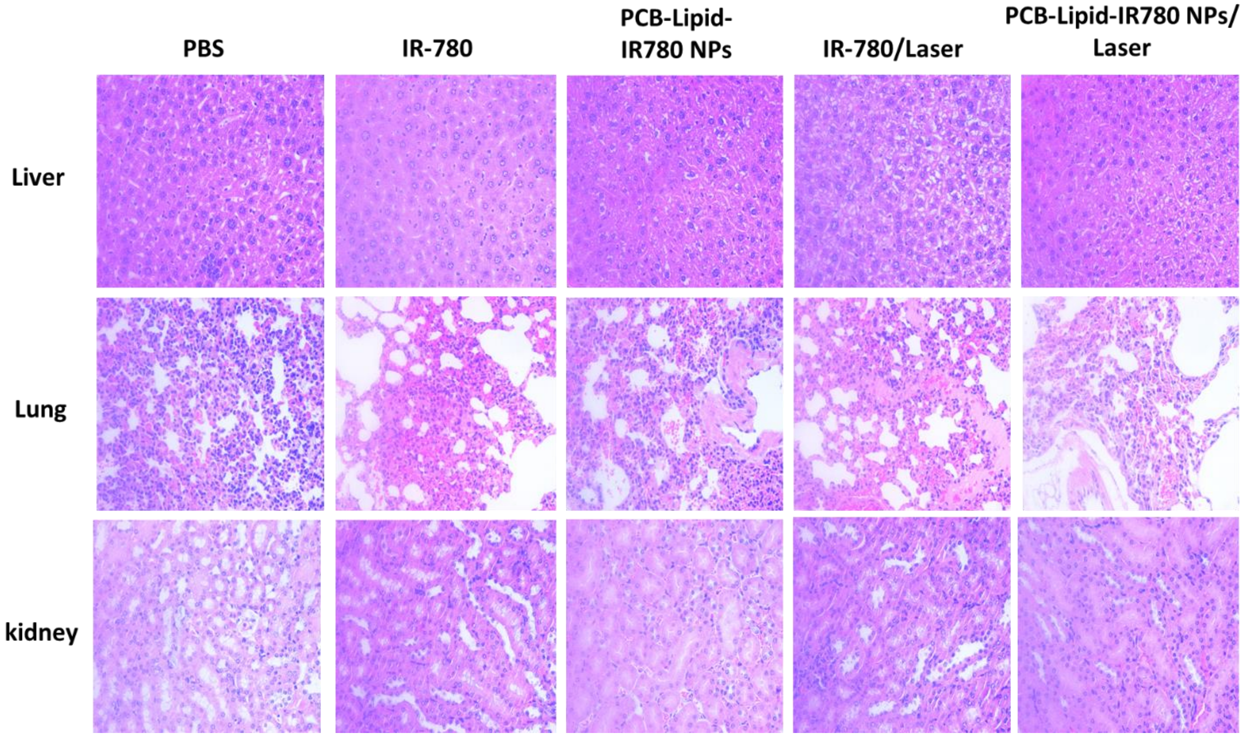

Figure S6. H&E staining of liver, lung, and kidney from PTT-treated TC-1 xenograft mouse models administered with PCB-lipid–IR-780.
